# Study on the Effect of Rare Earth Oxide Addition on the Microstructure and Properties of Ni60/WC-Ni Coatings Prepared by Laser Cladding

**DOI:** 10.3390/ma16237263

**Published:** 2023-11-21

**Authors:** Yanchun Chen, Xu Huang, Jibin Jiang, Guofu Lian, Changrong Chen

**Affiliations:** 1 Fujian Key Laboratory of Intelligent Machining Technology and Equipment, Fujian University of Technology, Fuzhou 350118, China; chenyc19980102@163.com (Y.C.); huangxu@fjut.edu.cn (X.H.); gflian@mail.ustc.edu.cn (G.L.); changrong.chen@fujt.edu.cn (C.C.); 2 Department of Mechanics, School of Mechanical and Automotive Engineering, Fujian University of Technology, Fuzhou 350118, China

**Keywords:** laser cladding, rare earth oxide, Ni-based composite coating, hardness, wear resistance

## Abstract

Rare earth oxides have been proven for their ability to refine grains and have high melting points. In this paper, different contents of rare earth oxide La_2_O_3_ were added into the Ni60/WC-Ni composite coating, in order to study its effect on the coating properties. SEM observation confirmed that the grain was refined significantly after the addition of La_2_O_3._ Energy Dispersive Spectroscopy (EDS) was applied to investigate the composition and X-Ray Diffraction (XRD) was used to measure the residual stress in the coating samples. In addition, the microhardness and wear resistance of the samples were tested. The results showed that the dilution ratio of coatings with different additions of La_2_O_3_ was in the range of 2.4 to 9.8%, and the sample with 1.0% addition of La_2_O_3_ exhibited the highest hardness of 66.1 HRC and best wear resistance with a wear volume of 9.87 × 10^6^ μm^3^, and the residual stress increased from 159.4 MPa to 291.0 MPa. This implies that the performance of the coating has been obviously improved after the addition of La_2_O_3_.

## 1. Introduction

Laser cladding technology applies high energy density laser beams to melt powder and forms a dense layer with metallurgical bonding to the substrate, which has a low dilution ratio and small thermal deformation. Laser cladding has been widely used in various applications such as the surface modification of metals and repair of components [1,2,3]. Besides the laser technology, the powders used for laser cladding also play an important role in the performance of the fabricated coatings. Recently, a single-powder coating can no longer meet the complex working conditions, and using mixed powder (for example, metallic powders mixed with ceramic powders) for laser cladding has become one of the research highlights [4,5]. Due to its excellent oxidation resistance and high hardness, Ni/WC composite powder is currently used in many industrial areas [6,7]. However, due to the different densities of metallic and ceramic powders, the heavy, hard phase such as WC would drop and precipitate at the bottom of the molten pool, which increases the possibility of coating fractures and leads to a lower wear resistance of the coating, while less hard phases remain in the upper area of the coating [8].

Rare earth oxides (REOs) as a head class of materials have generated global interest in modern technology. They have applications in corrosion protection, thermal barrier coating, hydrophobic coating, catalytic reactions, refrigeration and environmental protection [9,10]. Studies show that the addition of a rare earth oxide such as La_2_O_3_ can improve the performance of laser cladding coatings significantly, while rare earth oxide is proven to be able to refine grains, reduce impurities and optimize the grain distribution of the cladding layer because of its high chemical activity [11,12]. On the other hand, it can act as a diffusion barrier by producing stable rare earth oxides or additional protective films, which significantly enhances the oxidation resistance of the alloy [13].

Han et al. [14] found that the addition of La_2_O_3_ in Mg cladding layers could reduce its porosity and improve the corrosion and wear resistance. Cheng et al. [15] found that La_2_O_3_ addition enhanced the high-temperature creep properties of Mo alloys and discovered a critical proportion of Mo-La_2_O_3_ alloys. Beyond the critical proportion, the fracture area of the probe could reduce by up to 90% when a dimple fracture occurred. Cui et al. [16] studied the effect of LaB6 doping on the microstructure of laser-cladded FeCoNiCrMo high-entropy alloy coating on a Ti_6_Al_4_V substrate. The results indicated that an appropriate content (2.0 wt%) addition of LaB_6_ could significantly reduce the cracking sensitivity of the coating, and the in situ synthesized La_2_O_3_ could refine the coating microstructure through a fine grain strengthening effect.

Previous research revealed that the addition of La_2_O_3_ in the cladding layers has a positive effect on the microstructure, which could significantly improve the mechanical properties and has a wide market prospect. In this paper, La_2_O_3_ was mixed into Ni/WC powders for laser cladding on 45-steel substrates to study the effect of La_2_O_3_ addition and its content on the microstructure and mechanical properties of the cladding layers.

## 2. Materials and Methods

### 2.1. Sample Preparation

The chemical composition of the substrate (45-steel, with dimension size of 40 mm × 20 mm × 10 mm) is shown in Table 1, in which Bal. means balance element. All substrates are washed with ethanol to remove any grease of dirt before experiment. Table 2 shows the chemical composition of the powders used for laser cladding, which is Ni60A with WC-Ni (40% of which are WN25 and WC-Ni). The percentage of rare earth oxide La_2_O_3_ is set as 0.5%, 1%, 1.5%, 2%, 2.5% and 3% in comparative experiment design, as shown in Table 3. Powders are mixed through mechanical ball milling and dried before laser cladding.

### 2.2. Laser Cladding Process

**Figure 1:**
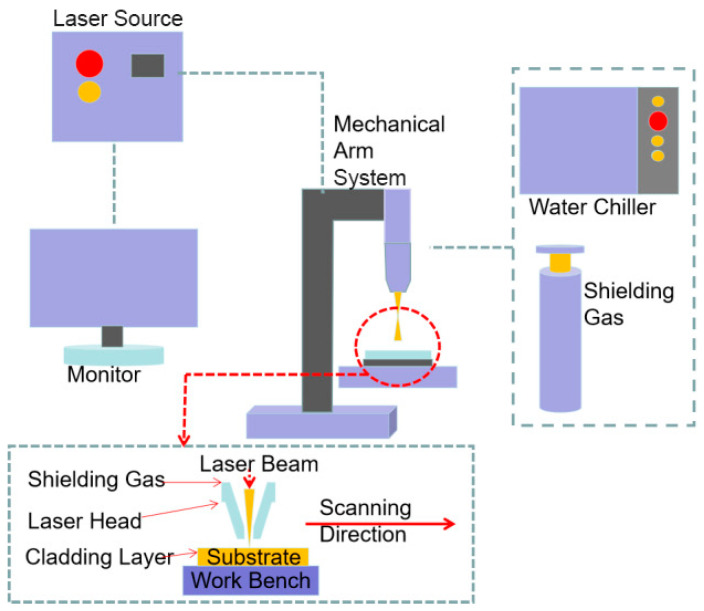
Schematic diagram of experimental equipment.

High-power fiber laser equipment (YLS-2500, IPG Photonics, Oxford, MA, USA) was employed for the laser cladding process. Figure 1 is a schematic diagram of the laser cladding process. Single-track laser cladding layers were fabricated on the substrate under the following parameters: 1.6 KW power, 5 mm/s laser scanning speed, 10 mm working distance, 45 rpm powder feeding rate and 55% overlapping rate. Argon was used as the shielding gas.

**Figure 2:**
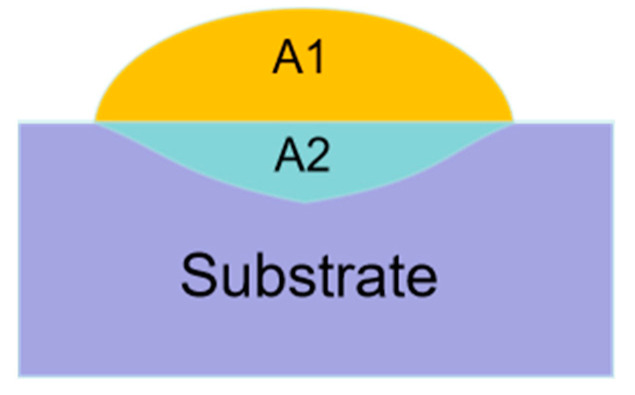
Schematic diagram of the sample cross-section.

A wire electrical discharge machining machine (WEDM) was used to cut the cladding samples into small pieces with dimension 10 mm × 6 mm × 3 mm. After grinding and polishing, they were etched for characterization. A scanning electron microscope (SEM, TM 3030 Plus, Oberkochen, Germany) with a configured energy dispersive spectroscopy (EDS, OXFORD Xplore, Oxford, UK) was used for microstructure and wear surface observation with extra-high tension at 10 kV and working distance at 12.7 mm. In addition, X-Ray Diffraction (XRD, Bruker D8 ADV ANCE, Karlsruhe, Geramny) was performed to study the phase composition of the coating with Cu Kα radiation at 95 mA and 40 kV and a scanning speed of 2.5°/min, ranging from 10° to 80°. Three-dimensional microscopy (KH-1300) was used to measure the cross-sectional area of each sample, and the dilution ratio could be calculated with the following simplified formula:

$$\eta =\frac{A_{2}}{A_{1}+A_{2}}\times 100\%$$

where ƞ stands for the dilution ratio, *A*_2_ is the area of the molten substrate, and *A*_1_ is the area of the cladding layer (as shown in Figure 2).

A microhardness tester (MVA-402TS) was employed in the experiment with a load of 9.8 N and holding time of 20 s. Hardness was measured 3 times at the same depth from the coating surface to the substrate. Residual stress was measured using XRD (Bruker D8 ADVANCE), the cutting samples were placed on the working table, and the measuring sensor acquired data from surface to substrate at a certain distance. Wear resistance was determined using a Burker (Ht-1000) testing machine, the friction pair of which was a hard alloy ball with 10 mm diameter. The wear experiment was conducted through linear reciprocation friction mode with a distance of 4 mm under a load of 35 N and duration of 30 min. After the wear test, all samples were cleaned with ethanol before further study. A white light interferometer (NV5000) was used to scan the three-dimensional morphology of the friction area and calculate the wear volume loss.

## 3. Results and Discussion

### 3.1. Macrostructure of the Cladding Layer

**Figure 3:**
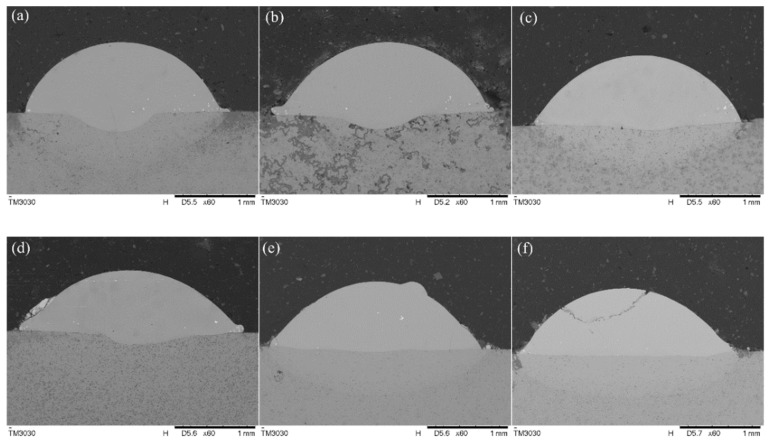
Cross section of the samples with different contents of La_2_O_3_: (**a**) 0.5 wt%; (**b**) 1.0 wt%; (**c**) 1.5 wt%; (**d**) 2.0 wt%; (**e**) 2.5 wt%; (**f**) 3.0 wt%.

Figure 3 shows the cross-section morphology of the samples with different La_2_O_3_ additions. It can be seen that there were numerous bright white particles, which were confirmed as WC. The WC ceramic particles with shape edges were prone to causing a stress concentration, making it difficult to bond tightly with the matrix. With the addition of La_2_O_3_, the shape edges of WC particles were rounded off, which improved the bonding condition with the matrix, and thus, more WC particles were retained in the cladding layers [17]. Additionally, the increase in La_2_O_3_ could reduce the wetting angle of the cross sections. It is more obvious when the La_2_O_3_ content is above 1.5%. The rare earth oxide has been proven to absorb the laser energy. As the content of La_2_O_3_ increases, the energy intake of the layers increases as well, which would enhance the fluidity of the molten pool. The defect of porosity is not obvious at the interface of the cladding layer in all of the samples, as a high-energy laser can fully melt the rare earth elements into the molten pool. La_2_O_3_ can serve as a surface-active substance to promote the microalloying process of the molten pool. When La_2_O_3_ reaches a supersaturated state, it forms compounds with the elements such as O, S, and Si in the cladding layer and floats up in the molten pool. The phenomenon of floatation reduces the decarburization of the hard phase WC in the coating, thereby suppressing the generation of porosity defects [18].

With the increasing La_2_O_3_ content in the coatings (for example 2.5 wt% and 3.0 wt%), small cracks were visible in some samples, as shown in Figure 3d (two microcracks on both sides) and Figure 3f (obvious crack in the upper area), which implies that there is a critical composition ratio of La_2_O_3_ in the alloys. Excessive rare earth elements would reduce the fluidity of the liquid alloys in the molten pool. The addition of excessive rare earth elements increased the absorption ratio of cladding materials, and as a result, the energy in the molten pool raised and released more WC particles. The carbon atoms decomposed from WC would react with oxygen elements in La_2_O_3_ and caused the generation of CO. The emission of CO caused a more severe splashing in the molten pool, which reduced the mass of the molten pool and led to a ratio change of Ni to WC. Due to the concentration of WC particles and the significant difference in the thermal expansion coefficient, huge residual stress remained after the solidification of the molten pool, which could lead to the crack of the coating. In addition, due to the big gap in thermophysical properties between La_2_O_3_ and the substrate, stress concentrations would easily occur around the hard second phases, which cause the initiation of the cracks in the coatings.

**Figure 4:**
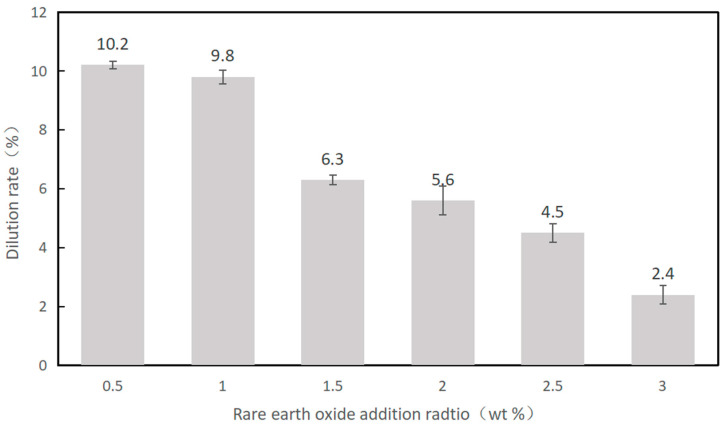
The dilution ratio of coatings with different La_2_O_3_ additions.

Figure 4 displays the calculation results for the dilution ratio of the samples listed in Figure 3. The dilution ratio of the samples was between 2.4 wt% and 11.1 wt% with minor fluctuations. The addition of La_2_O_3_ in the mixed powders could absorb more laser energy during the cladding process, which enlarged the molten pool and improved the fluidity of the molten pool. As a comparison, samples with 1.0 wt% La_2_O_3_ exhibit a better dilution ratio.

### 3.2. Microstructure of the Cladding Layer

**Figure 5:**
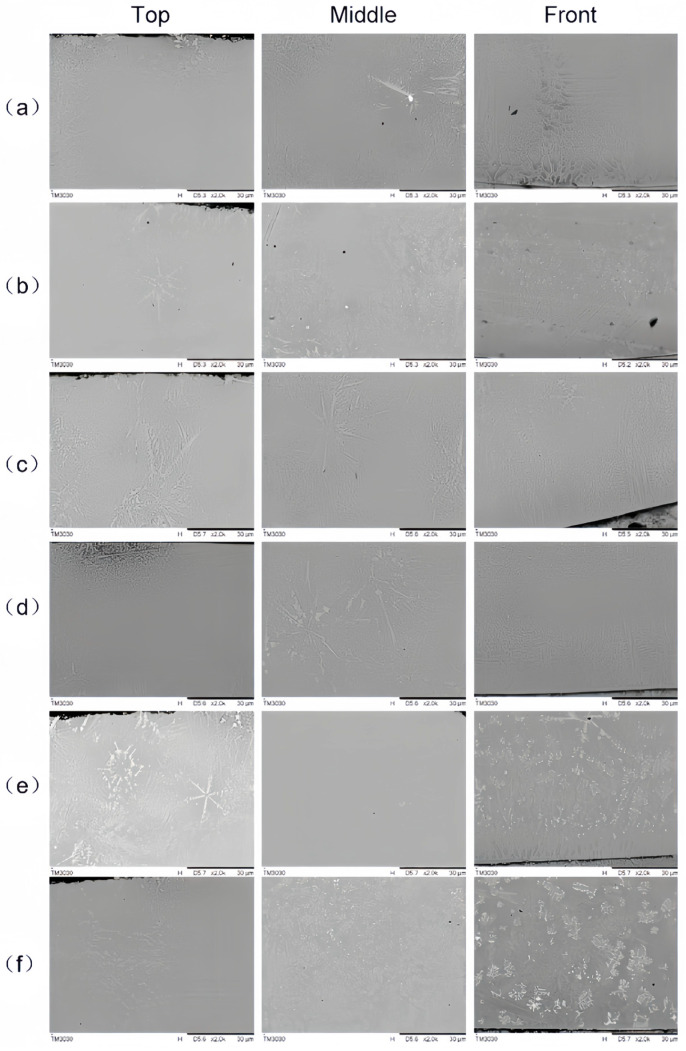
Microstructure (Cross section) of the cladding layers with different contents of La_2_O_3_: (**a**) 0.5 wt%; (**b**) 1.0 wt%; (**c**) 1.5 wt%; (**d**) 2.0 wt%; (**e**) 2.5 wt%; (**f**) 3.0 wt%.

Figure 5 shows the microstructure of the coatings with different additions of La_2_O_3_. As can be seen, the cladding layer is divided into three areas from top to front, namely, the equiaxed grain area, the dendrite area and the planar grain area.

**Figure 6:**
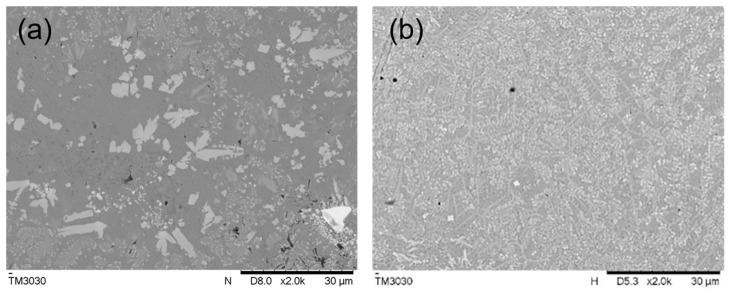
Microstructure of the coating in the middle area: (**a**) coating without La_2_O_3_; (**b**) coating with 1.0 wt% La_2_O_3_.

Figure 6 shows the microstructure of middle area of the sample without rare earth elements and the sample with 1.0 wt% addition of La_2_O_3_. With the addition of rare earth elements, the coarse dendrites became smaller and closer, the grains distributed in the dendritic area also became finer, which was caused by the high concentration of rare earth elements at the solid–liquid interface, and then increased the undercooling level. At the same time, the fine dendrites were easily melted and broken after the absorption of high laser energy, which produced more new grains and increased the density of grains [19].

As shown in Figure 5, the dendrites in the coating became coarse when the content of rare earth elements was over 1.0 wt%, as more needle-like dendrites transferred to equiaxed dendrites. The excessive rare earth elements reacted with oxygen and silicon elements in the coating and formed a few impurities with high melting points, which reduced the fluidity of the molten pool and suppressed the growth of the grains [20].

**Figure 7:**
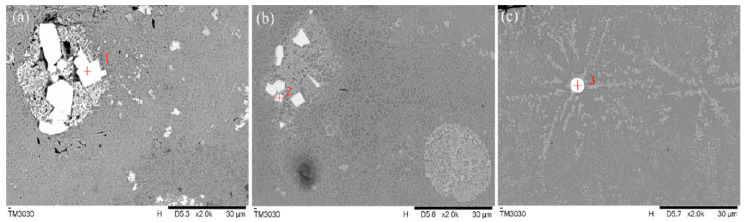
The morphology for WC particles with addition of La_2_O_3_; (**a**) 0.5%, (**b**) 1.5%, (**c**) 2.5% La_2_O_3._

Figure 7 shows the morphology for WC particles when La_2_O_3_ was added. Obvious differences in the hard phase WC were observed according to the La_2_O_3_ content. For samples with less La_2_O_3_ addition (0.5 wt%, as shown in Figure 7a), there are still inclusions in the coating. However, the convection in the molten pool is insufficient for WC particles to melt, and therefore, the WC particles are still larger with obvious edges, and the distribution is uneven [21]. Figure 7b,c show the morphologies of coating samples with 1.5 wt% and 2.5 wt% La_2_O_3_. With the increasing La_2_O_3_ content, the WC particles become smaller and more rounded. However, the rare earth oxides tend to aggregate at the surface of the WC particles, in particular, at the sharp edges, where the surface free energy is high. The concentration of the rare earth elements could accelerate the melting and decomposition of the particles, which contributes to the bonding of hard WC particles with other powders and makes the cladding layer much denser.

Table 4 shows the EDS results of the points marked (1–3) in Figure 7. In consideration of the SEM images of the coating, the La_2_O_3_ particles with a melting point of 2315 °C cannot be melted completely in the molten pool. The unmelted particles were concentrated in the surrounding of the grains, which inhibits the growth of the grain. Additionally, the decomposed La element in La_2_O_3_ has a large atom radius and high chemical activity. Some of them gathered at the grain boundaries, which suppressed the growth of the grains due to the drag effect. Table 4 also confirms the existence of the rare earth element La and the formation of a new W/C composition in the cladding layer.

### 3.3. Microhardness of the Cladding Layer

**Figure 8:**
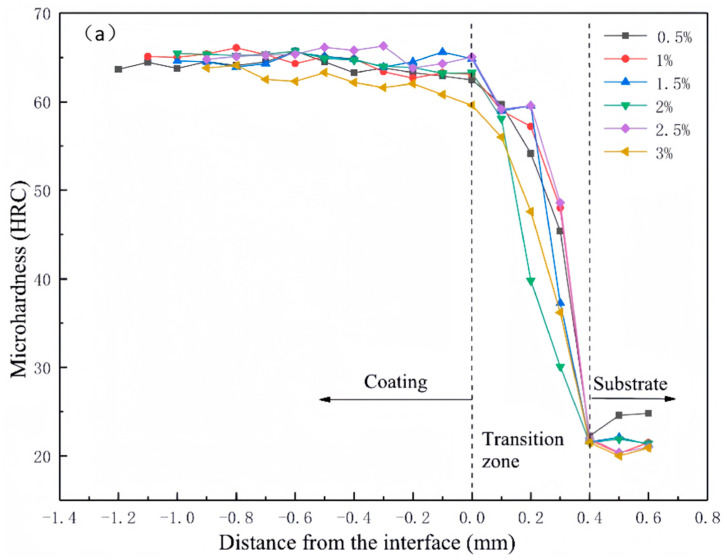

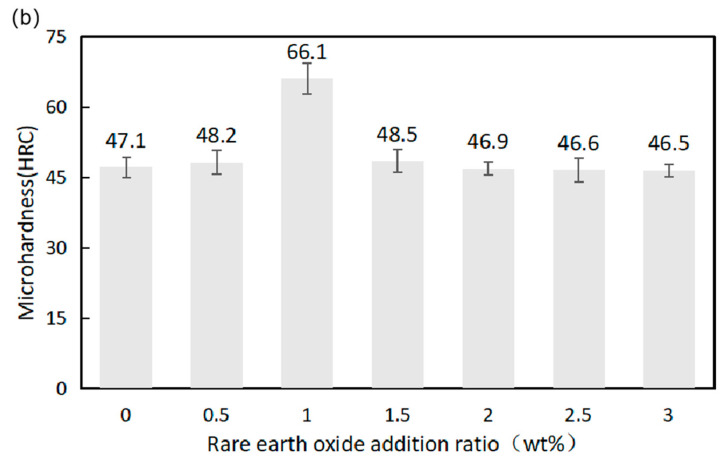
Microhardness results of the samples: (**a**) hardness development according to the coating depth; (**b**) average microhardness of coatings with different content of La_2_O_3._

Figure 8 shows the microhardness distribution of the cladding layers with different La_2_O_3_ content. As seen, the microhardness of the cladding layer reduced significantly with increasing depth. The cladding layer with 1.0 wt% La_2_O_3_ exhibited the highest microhardness of 66.1 HRC and was well-distributed, which was caused by the even distribution of WC after the addition of La_2_O_3_. Meanwhile, rare earth elements can eliminate the effects of harmful elements in the molten pool, such as O, S, and Si, which is beneficial for the improvement of the coating microhardness.

However, excessive rare earth oxides in the coating (such as the sample with 3.0 wt% La_2_O_3_) could also reduce the coating microhardness, while excessive concentrations of rare earth oxides would cause an increase in the melting and burning of the hard WC particles. In addition to that, the excessive addition of La_2_O_3_ led to the splashing of the cladding layer, resulting in a mass loss of hard phases and coarse grains. The impurities remained in the molten pool after cooling, causing a decrease in the coating hardness.

### 3.4. Wear Resistance of the Cladding Layer

**Figure 9:**
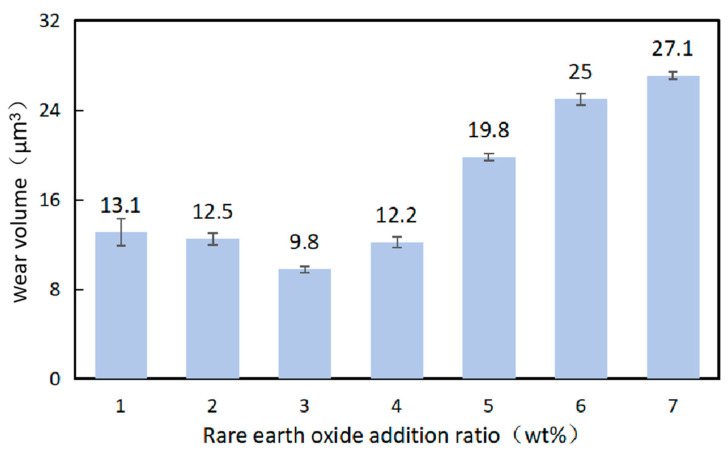
Schematic diagram of the sample wear volume according to the addition of La_2_O_3._

Figure 9 shows the wear volume of the coatings with different contents of La_2_O_3_. With increasing La_2_O_3_ in the coating, the wear volume of the samples firstly decreased and then increased, indicating that the addition of La_2_O_3_ could influence the wear resistance of the coatings, which with an appropriate proportion. It can be seen from Figure 8 that the coating sample with 1.0 wt% La_2_O_3_ exhibited the best wear resistance with a wear volume of 9.87 × 10^6^ μm^3^. Previous research has shown that the solid solubility of rare earth oxide in metals is very low, which causes the segregation of rare earth oxides at the grain boundaries and strengthens them, and as a result, the mobility of the dislocations at the grain boundaries decreased [22]. At the same time, the toughness of the coatings increased when the grains were refined, and thus, the wear resistance improved.

As shown in Figure 8, the coating sample with 3.0 wt% La_2_O_3_ displays the worst wear resistance. An excessive addition of La_2_O_3_ in the coating would accelerate the burning of WC particles, resulting in a decrease in the hard phase in the coatings. In addition, the fluidity of the molten pool reduced when the content of La_2_O_3_ was too high, leading to the concentration of inclusions and pores in the coating and an uneven distribution of WC, and thus reduced the coating wear resistance.

### 3.5. Residual Stress of the Cladding Layer

Residual stress represents the distribution of stress in a material that is not at equilibrium, which is usually caused by the elimination of external stress of uneven temperature fields. Laser cladding is a fast heating and cooling process, which generates a high temperature gradient and thus high residual stress in the coating. Residual stress has significant effects on the properties and performance of components, which usually causes the initiation of internal cracks and the failure of components. In the case of laser cladding samples, the residual stress remaining high would lead to the coating detachment [23]. As shown in Figure 3, transverse cracks are found in the samples with the addition of rare earth oxides after the friction and wear experiments, which is caused by the release of residual stress during the test.

**Figure 10:**
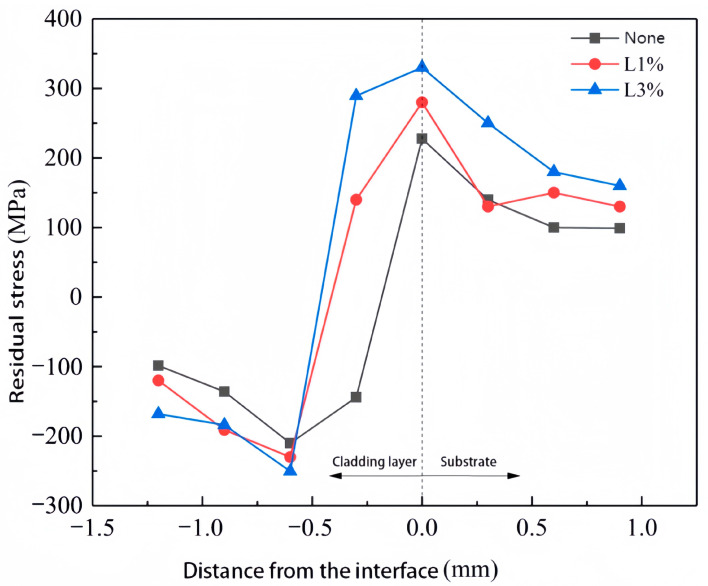
Residual stress distribution in the samples with different contents of La_2_O_3._

Figure 10 shows the residual stress of samples with different La_2_O_3_ in cooperation with the sample without the addition of La_2_O_3_, in which the maximum residual stress occurs in the sample with 3.0 wt% addition of La_2_O_3_ and the minimum residual stress occurs in the sample with 1.0 wt% addition of La_2_O_3_ (based on the results shown in Figure 8). It can be seen clearly that the distribution of the residual stress in the cladding layer is uneven, and the peak residual stress always appears at the bonding area of the coating and substrate. The reason for this is that the fast heating and cooling laser cladding process improved the diversity of the expansion and contractility ability between the coating material and substrate. According to Figure 10, the maximum residual stress of the sample with 3.0 wt% La_2_O_3_ reached 325 MPa and the sample with 1.0 wt% La_2_O_3_ reached 291 MPa. The results show that, under the same conditions, the maximum residual stress in samples with the addition of La_2_O_3_ is larger than in those without La_2_O_3_. The addition of rare earth oxides can strengthen the absorption of the laser energy. When the energy in the molten pool increases, the temperature difference during the heating and cooling process between the coating and substrate will rise as well, which leads to larger residual stress.

## 4. Conclusions

The ceramic coating on steel refers to the application of a thin layer of ceramic material onto the surface of the steel. This coating acts as a barrier that provides protection against wear, corrosion, and high temperatures. The ceramic coating enhances the durability and longevity of the steel by reducing friction, improving the thermal stability, and providing a smooth and hard surface. The application of ceramic coatings on steel is widely used in the automotive, aerospace and industrial sectors. A ceramic coating over steel surface and its properties and applications have been reviewed in this work [24].

In this paper, different contents of La_2_O_3_ have been added into the Ni60/WC-Ni composite coating to study its effect on the coating properties such as microhardness and wear resistance. The following conclusions can be drawn:

(1) With the addition of La_2_O_3_, the amount of hard WC particles obviously increased. La_2_O_3_ has the effect of improving the wetting property of Ni60 powder and WC hard particles, which can reduce the detachment of WC particles in the cladding layer.
(2) The properties of the coating surface improved significantly after the addition of La_2_O_3_. However, an excessive addition of La_2_O_3_ reduced the wetting angle of the coating and made the coating surface flatter, especially when the content of La_2_O_3_ was over 1.5 wt%. At the same time, rare earth oxides have the ability to absorb the laser energy. With the increasing content of La_2_O_3_, more energy was absorbed into the molten pool, which led to a lower heat dissipation efficiency. As a result, the fluidity of the molten pool increased and expanded to both sides.
(3) The dilution ratio of the samples after the addition of La_2_O_3_ was between 2.4 wt% and 11.1 wt%, with small fluctuations. The sample with 1.0 wt% La_2_O_3_ addition reached the highest microhardness of 66.1 HRC.
(4) Samples with 1.0 wt% La_2_O_3_ exhibited the best wear resistance, with the smallest wear volume of 9.87 × 10^6^ μm^3^ and the largest residual stress of about 291 MPa.

## Figures and Tables

**Table 1:** Chemical composition of the 45-steel substrate (wt%).

Element	Fe	C	Si	Ni	Cu	Cr	Mn
Content	Bal.	0.42~0.50	0.17~0.37	≤0.30	≤0.25	≤0.25	0.50~0.80

**Table 2:** Chemical composition of Ni60A powders (wt%).

Element	Ni	Cr	Fe	Si	B	C
Content	Bal.	16.0	≤5.0	4.0	3.2	0.9

**Table 3:** Experiment design of La_2_O_3_ addition into the coatings (wt%).

Sample Group	1	2	3	4	5	6
Content	0.5	1.0	1.5	2.0	2.5	3.0

**Table 4:** EDS analysis of the samples with addition of La_2_O_3_.

Element Content (%)	Point 1	Point 2	Point 3
C	39.3	37.1	45.4
Cr	1.0	1.1	1.2
Ni	0.0	0.5	0.8
La	0.7	1.5	0.6
W	58.1	57.9	51.1
Si	0.8	1.2	0.8
B	0.1	0.1	0.1

## Data Availability

The data presented in this study are available on request from the corresponding author.

## References

[1] Gong X., Wang J., Feng H. (2019). Lateral powder transport model with Gaussian distribution in laser cladding. Int. J. Adv. Manuf. Technol..

[2] Lu Y., Huang G., Wang Y., Li H., Qin Z., Lu X. (2018). Crack-free Fe-based amorphous coating synthesized by laser cladding. Mater. Lett..

[3] Zhu L., Xue P., Lan Q., Meng G., Ren Y., Yang Z., Xu P., Liu Z. (2021). Recent research and development status of laser cladding: A review. Opt. Laser Technol..

[4] Wang H.-Z., Cheng Y.-H., Yang J.-Y., Wang Q.-Q. (2020). Microstructure and properties of laser clad Fe-based amorphous alloy coatings containing Nb powder. J. Non-Cryst. Solids.

[5] Yang S., Meng Q., Geng L., Guo L., Wu L. (2007). Ni–TiC coating deposited on Ti–6Al–4V substrate by thermal spraying and laser remelting of Ni-clad graphite powder. Mater. Lett..

[6] Hu Z., Li Y., Lu B., Tan N., Cai L., Yong Q. (2022). Effect of Wc Content on Microstructure and Properties of High-Speed Laser Cladding Ni-Based Coating. Opt. Laser. Technol..

[7] Hu Y., Wang Z., Pang M. (2022). Effect of WC content on laser cladding Ni-based coating on the surface of stainless steel. Mater. Today Commun..

[8] Du M., Wang L., Gao Z., Yang X., Liu T., Zhan X. (2022). Microstructure and element distribution characteristics of Y_2_O_3_ modulated WC reinforced coating on Invar alloys by laser cladding. Opt. Laser Technol..

[9] Das A.K. (2022). Effect of rare earth oxide additive in coating deposited by laser cladding: A review. Mater. Today Proc..

[10] Zhang Z., Yang Q., Yu Z., Zhang T., Jing J. (2023). Microstructure and performance of Ti-based wear-resistant laser cladding coatings with rare-earth oxides. Mater. Charact..

[11] Cao Q., Fan L., Chen H., Hou Y., Dong L., Ni Z. (2022). Wear behavior of laser cladded WC-reinforced Ni-based coatings under low temperature. Tribol. Int..

[12] Wang W., Yu Q., Liu X., Huang K., Mi J., Hao L., Lu Z. (2022). Microstructure and deuterium resistance of Al_2_O_3_/Y_2_O_3_ composite coating with different annealing atmospheres. Rare Met..

[13] Yu L., Zhang Y., Fu T., Wang J., Cui K., Shen F. (2021). Rare Earth Elements Enhanced the Oxidation Resistance of Mo-Si-Based Alloys for High Temperature Application: A Review. Coatings.

[14] Han J., Yu Y., Yang J., Xiaopeng L., Blawert C., Zheludkevich M.L. (2022). Corrosion and wear performance of La_2_O_3_ doped plasma electrolytic oxidation coating on pure Mg. Surf. Coat. Technol..

[15] Cheng P.M., Chong Y., Zhang P., Zhang J., Wang H., Kuang J., Liu G. (2022). Enhancing the high-temperature creep properties of Mo alloys via nanosized La_2_O_3_ particle addition. J. Mater. Sci. Technol..

[16] Cui C., Wu M., He R., Jie D., Gong Y., Miao X. (2023). Effect of LaB_6_ doping on the microstructure, microhardness and corrosion behavior of laser cladded FeCoNiCrMo coating on Ti6Al4V. Surf. Coat. Technol..

[17] Wang H., Sun Y., Qiao Y., Du X. (2021). Effect of Ni-coated WC reinforced particles on microstructure and mechanical properties of laser cladding Fe-Co duplex coating. Opt. Laser. Technol..

[18] Zhang H., Pan Y., Zhang Y., Lian G., Cao Q., Que L. (2022). Microstructure, toughness, and tribological properties of laser cladded Mo_2_FeB_2_-based composite coating with in situ synthesized WC and La_2_O_3_ addition. Surf. Coat. Technol..

[19] Hou Q., Huang Z., Gao J. (2007). Effects of Y_2_O_3_ on the microstructure and wear resistance of cobalt-based alloy coatings deposited by plasma transferred arc process. Rare Metals.

[20] Sktani Z.D.I., Rejab N.A., Rosli A.F.Z., Arab A., Ahmad Z.A. (2021). Effects of La_2_O_3_ addition on microstructure development and physical properties of harder ZTA-CeO_2_ composites with sustainable high fracture toughness. J. Rare Earths.

[21] Tlili M., Amor M., Gabrielli C., Joiret S., Maurin G., Rousseau P. (2002). Characterization of CACO_3_ hydrates by micro-Raman spectroscopy. J. Raman Spectrosc..

[22] Zhang Z., Yang Q., Yu Z., Wang H., Zhang T. (2022). Influence of Y_2_O_3_ addition on the microstructure of TiC reinforced Ti-based composite coating prepared by laser cladding. Mater. Charact..

[23] Wang Q., Shi J., Zhang L., Tsutsumi S., Feng J., Ma N. (2020). Impacts of laser cladding residual stress and material properties of functionally graded layers on titanium alloy sheet. Addit. Manuf..

[24] Akhil U.V., Radhika N., Rajeshkumar L., Sivaswamy G. (2023). A Comprehensive Review on Ceramic Coating on Steel and Centrifugal Thermite Process: Applications and Future Trends. J. Bio-Tribo-Corros..

